# Assessment of antigenemia among children in four hotspots of filarial endemic districts of Nepal during post-MDA surveillance

**DOI:** 10.1186/s41182-023-00538-4

**Published:** 2023-08-24

**Authors:** Pramod Kumar Mehta, Mahendra Maharjan

**Affiliations:** Central Department of Zoology, Institute of Science and Technology, Tribhuvan University, Kirtipur, Nepal

**Keywords:** Circulating filarial antigen (CFA), Lymphatic filariasis, Mass drug administration, Transmission assessment survey, *W. bancrofti*

## Abstract

**Background:**

Sixty-three out of 77 districts reported lymphatic filariasis (LF) endemic in Nepal. Mass drug administration (MDA) with diethylcarbamazine (DEC) and albendazole (ALB) treatment program was continued for 6 to 11 rounds in these districts. Nepal government has stopped the MDA program based on the transmission assessment survey (TAS) report of 2014 and 2018 indicating *Wuchereria bancrofti* antigenemia prevalence < 2%. But the persistence of low levels of the circulating filarial antigen (CFA) in some foci of four endemic districts of Central Nepal, i.e., 0.4% in Dhading, 0.7% in Mahottari, 0.21% in Lalitpur and 1.2% in Bara district could responsible for enhancing the risk of infection resurgence. Hence the present study was designed to assess antigenic prevalence using Filariasis Test Strip (Alere, Scarborough ME) in children born after MDA in hotspot areas of four endemic districts of Central Nepal.

**Results:**

The present study covers 70% children of the eligible population. The result revealed significantly high CFA prevalence in hotspots of Mahottari district belonging to the Terai region and Dhading district belonging to the hilly region, i.e., 13% and 10%, respectively, compared to baseline prevalence and TAS report. While in Lalitpur district and Bara district CFA prevalence was still found to be less than 2%. A higher number of MDA rounds covered in hotspots were found significantly associated with the low antigenic prevalence of *W. bancrofti*. Whereas median treatment coverage and inter-quartile range (IQR) in study districts were not found significantly associated with CFA prevalence. Although the clinical manifestation of hydrocele (1%) was found in all four study districts, it was not due to the *W. bancrofti* infection.

**Conclusions:**

Two hotspot regions, one each from the Terai (Mahottari) and hilly (Dhading) districts were found highly prevalent with CFA and significantly associated with the number of MDA rounds but were not associated with treatment coverage and IQR. Higher CFA prevalence was observed in hotspots where baseline prevalence was high together indicating that rounds of MDA program need to be extended further in these hotspot regions of endemic districts.

**Supplementary Information:**

The online version contains supplementary material available at 10.1186/s41182-023-00538-4.

## Background

Lymphatic filariasis (LF) is a neglected tropical disease found in more than 80 tropical and sub-tropical countries [1]. Approximately 51.4 million people are infected with filariasis in 50 different countries, whereas 859 million people are at risk of infection globally [2].

The disease is caused by a group of filarial nematodes: *Brugia malayi*, *B. timori*, and most commonly *Wuchereria bancrofti* [3]. Based on the appearance of microfilaria (mf) in peripheral blood, parasites are found in periodic and sub-periodic physiological races. The *W. bancrofti* with a given physiological race is transmitted by three species of mosquitoes belonging to the genera *Anopheles*, *Culex,* and *Aedes,* while *Brugia* species are transmitted by *Mansonia* mosquito vector carrying third-stage infective larvae [4]. More than 90% of LF infections all over the world are transmitted by a single vector species, *Culex quinquefasciatus* [5]. Adult worms of these parasites live in lymph vessels and lymph nodes for four to six years in humans. Infections of these parasites cause chronic clinical manifestations such as hydrocele, lymphedema, chyluria, and adenolymphangitis attacks [6]. Chronic clinical manifestation of this disease impacts long-term suffering morbidity along with high social stigma and economic burden to individuals as well as communities [7–10]. Even though the disease is not fatal, it is ranked as the second leading cause of disability [11, 12] and imposes a heavy burden on the healthcare infrastructure in endemic areas [13].

Three Asian countries, India, Indonesia, and Bangladesh as well as the African country Nigeria contribute about 70% of the infection Worldwide [14]. Lymphatic filariasis was identified as one of the six parasitic diseases which could be potentially eradicated [14]. Preventive chemotherapy using diethylcarbamazine (DEC) and albendazole is recommended for interrupting the transmission of LF. In mass drug administration, all eligible people of endemic districts were given a single dose of two drugs (DEC and albendazole) together once a year for at least 5 years. Although the prevalence of LF significantly declined from 2000 to 2018, the LF elimination target by 2020 seems not possible to achieve [15]. In 2021, WHO estimated over 882 million people remained threatened in 44 countries with LF worldwide hence LF elimination target was set for 2030 [16].

A total of 63 districts out of 77 are potentially endemic for lymphatic filariasis in Nepal, while 14 districts are in the mountainous regions and unlikely to be endemic [17, 18]. Concerning the WHO target, the Nepal government has formulated National Task Force (2003–2020 AD) and launched a global program to eliminate lymphatic filariasis (GPELF) in 2003 [19]. Using mass drug administration (MDA), the program aimed to interrupt the transmission of microfilaremia in the community by 2020. National Taskforce MDA was started in 2007. In Bara, 11 rounds of MDA were conducted from 2007 to 2022, whereas in Mahottari and Dhading districts six rounds each were conducted. In the Lalitpur district, MDA was started in 2010 and stopped in 2017 with eight rounds of the MDA program. However, there was a cluster of antigen-positive cases found and these antigen-infection persistent sentinel sites of four districts were considered as hotspots. Therefore, the present study was carried out among the children born after the MDA program launched in hotspots of four endemic districts to monitor the risk of resurgence of new infection.

## Methods

### Study areas

During the year 2017–2018, a transmission assessment survey was carried out by the epidemiology and disease control division (EDCD) to assess the impact of mass drug administration in endemic districts of Central Nepal. The CFA prevalence ranged from 0% to 1.2% in the sentinel sites of each district so the maximum antigen-positive sentinel sites of four districts were selected for the current study. Areas with persistent infection were considered “hotspots”. Four hotspot areas from each of the four endemic districts, two districts from the hilly region (Lalitpur and Dhading), and two districts from the Terai region (Bara and Mahottari) were selected based on the TAS report 2018. Children born after 2007 in Bara, Mahottari, and Dhading districts and after 2010 in Lalitpur district were involved in an antigenemia survey.

#### Lalitpur district

A total population of Lalitpur district within the Kathmandu valley was 5,13,200 [20]. Eight rounds of annual MDA were completed in this district between 2010 and 2017 (Table 1). Two LF hotspot villages Bungmati of Lalitpur metropolitan city and Dhukuchhap of Godawari municipality were selected purposively.

**Table 1:** Reported treatment coverage during diethylcarbamazine and albendazole MDA intervention in selected endemic districts of Central Nepal

Region	Districts	Total population	No. of MDA rounds (% coverage)											IQR	Median coverage of MDA	*P*-value
			1	2	3	4	5	6	7	8	9	10	11			
Hilly	Lalitpur	548,401	68.3	67.9	*40.1*	*45.2*	*57.9*	*58.1*	78.2	64.6	–	–	–	19.8	61.4	0.004*
	Dhading	322,751	81.8	77.9	80.7	80.8	78.2	89.2	–	–	–	–	–	5.0	80.8	
Terai	Bara	743,950	82.6	84.7	82.9	86.4	77.1	80.7	82.6	71.4	81.8	82.9	84.9	4.0	82.6	0.039*
	Mahottari	705,838	83.5	89.7	80.1	86.7	87.8	92.3	–	–	–	–	–	7.6	87.3	

In italics, coverage not meeting the 65% epidemiological coverage threshold, “–” symbols indicate MDA was not implemented

MDA, mass drug administration; IQR, inter-quartile range

*P*-values with the “*” symbol were statistically significant

*P*-values shown in the results column are for differences compared to the median coverage of MDA

#### Dhading district

This district is another hilly district of Central Nepal. Tripurasundari rural municipality with a total population of 22,960 was selected as a hotspot area of this district [20]. In this district, MDA was started in 2007 and six rounds were completed between 2007 and 2013 (Table 1).

#### Bara district

This district lies in the Terai region of Central Nepal. Ammadar and Khairawa villages of Jeetpur sub-metropolitan city were selected as hotspots of this district where 11 rounds of MDA had been completed between 2007 and 2022 (Table 1).

#### Mahottari district

**Fig. 1:**
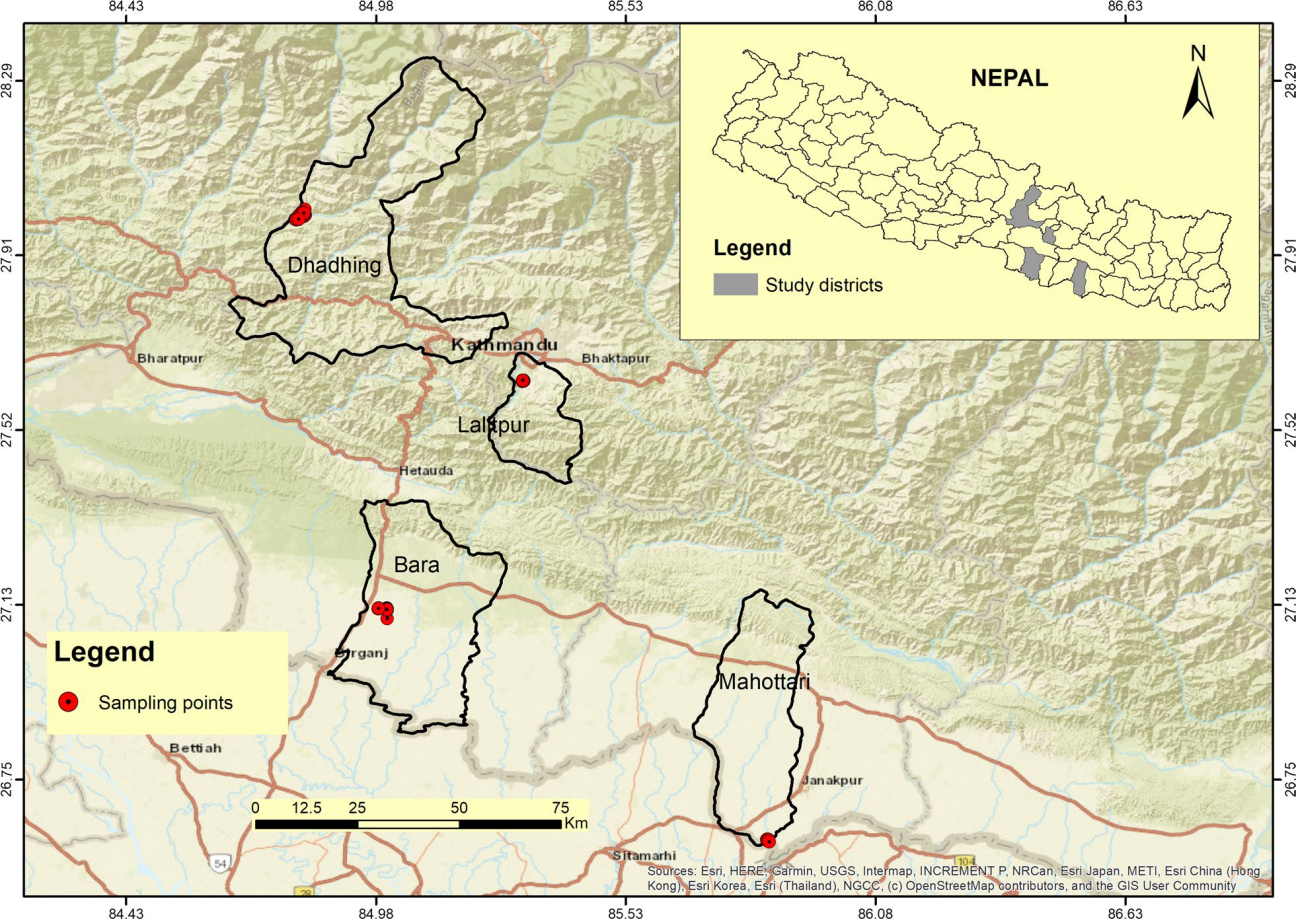
The map shows four filarial endemic districts with the approximate location of sampling points with red circles that were surveyed in 2019–2022

Matihani Municipality of this district was selected as one of the LF hotspot regions from the Terai region. In this district, six rounds of MDA had been completed between 2007 and 2013 (Table 1) (Fig. 1).

### Study population and sampling

A total of 724 households (hh) from identified hotspots of Lalitpur district (186 hh), Dhading district (174 hh), Bara district (141 hh), and Mahottari district (223 hh) with children born after MDA were purposely selected. Out of 1117 eligible populations in the study areas, 791 children of age groups 5–15 participated to the study based on their consent and availability.

### Antigenemia testing for human subjects

A circulating filarial antigen (CFA) was detected using Filariasis Test Strip (FTS, Alere, Scarborough ME) by fingers prick method. Briefly; using a plastic micropipette, 75 μL of blood was drawn and placed in the FTS sample application pad, and a single operator read the FTS for 10 min. The FTS results were scored semi-quantitatively. Each of the strips was labeled with patient ID, date, and result score. Test procedure recommended by the manufacturer was followed (Additional file 1: Fig. S1).

### Data analysis

Data were entered in Excel spreadsheets (Microsoft Excel 2007) and subsequently analyzed using Minitab 17 version 19.2.0. The results of FTS, presence of hydrocele or elephantiasis, and demographic characteristics were compared by using the Chi-square test and Fisher’s exact test while *p*-value ≤ 0.05 were considered statistically significant. The lower and upper limits of the 95% CI for the prevalence of CFA were calculated. Mann–Whitney *U* test was performed to examine the difference between the median treatment coverage.

## Results

A total of 1117 children were found eligible from selected hotspot regions of the four districts. Among them, 791 were included in this study. The most common reasons for individuals being missed in this survey (*n* = 326) were due to refusal to participate (62.5%) followed by being outside the home (22.5%) and being at school (15%). Overall 70% were sampled from both sexes covering the age group of 5 to 15 years. The maximum eligible population was covered from Dhading and Bara districts for the antigenic survey. The average mean age was 9.2 years and sex-wise 55% of males were involved (Table 2).

**Table 2:** Demographic characteristics of eligible samples and CFA prevalence of lymphatic filariasis in selected hotspot districts of Central Nepal

Demographic characteristics		Eligible population	Sampled population (%)	No. of CFA positives (%) (95% CI)	*P*-value
Total		1117	791 (70.8)	49 (6.2) (4.6–8.1)	–
Gender	Male	615	432 (70.24)	28 (6.5) (4.4–9.2)	NS
	Female	502	359 (71.51)	21 (5.9) (3.7–8.8)	
Age group (years)	5–9	652	436 (66.87)	24 (5.5) (3.6–8.1)	NS
	10–15	465	355 (76.34)	25 (7.1) (4.6–10.1)	
Hilly	Lalitpur	375	176 (46.93)	1 (0.6) (0.0–3.1)	< 0.001
	Dhading	208	202 (97.12)	20 (9.9) (6.2–14.9)	
Terai	Bara	227	211 (92.95)	2 (1.0) (0.1–3.4)	< 0.001
	Mahottari	307	202 (65.80)	26 (12.9) (8.6–18.3)	

CI, confidence interval, CFA, circulating filarial antigen, NS, non-significant

Interventions undertaken in hotspots of selected districts are presented in Fig. 1. A comparatively higher number of MDA rounds were completed in the Bara district of the Terai region followed by the Lalitpur district of the hilly region. The result is well correlated with the reduced antigenic prevalence. Median treatment coverage was significantly high in Dhading and Mahottari districts compared to other districts of the same region (Table 1), although the antigenic prevalence was not reduced (Table 2). Inter-quartile range (IQR) was comparatively high in the Mahottari district of the Terai region but the antigenic prevalence was not reduced; whereas, in the Dhading district of the hilly region, comparatively less IQR correlated with increased antigenic prevalence. The result indicated an insignificant association of median treatment coverage and IQR with the antigenic prevalence (Tables 1 and 2).

The upper confidence limit of CFA prevalence was greater than 2% in all four studied districts indicating a risk of a possible resurgence of Lf new infection. In general, CFA prevalence in children was not found significantly associated with age and gender (Table 2). Few hydrocele cases (8 of 791) were identified during the survey but no individuals were found with elephantiasis. None of the individuals with hydrocele was found to have CFA positive.

**Fig. 2:**
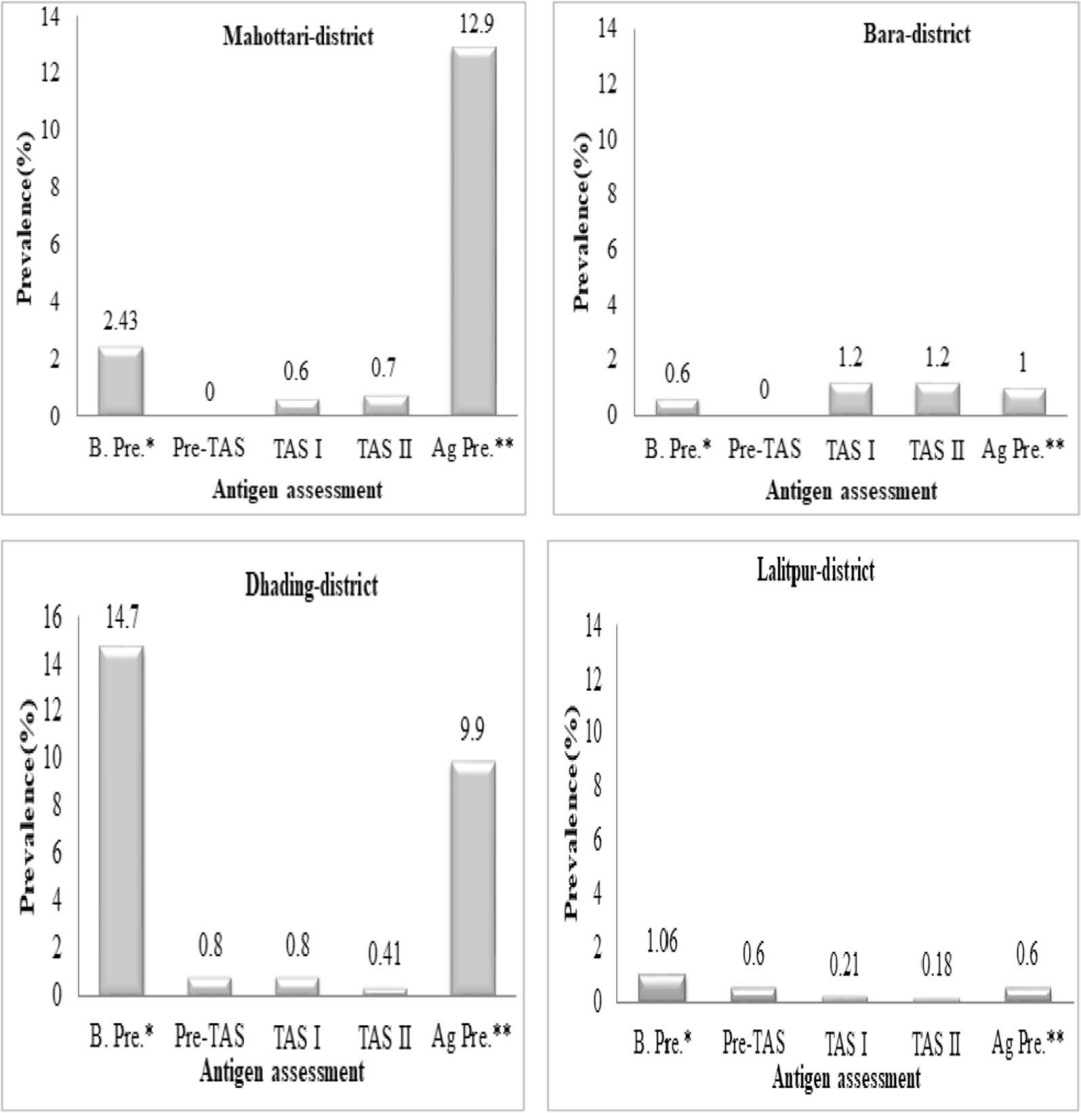
Trends of the prevalence of LF antigen (LF positive cases). *B. Pre., symbols indicate baseline prevalence. **Ag Pre., symbols indicate antigen prevalence

The current trends of the CFA positivity rate seem to be well correlated with baseline prevalence. Although all the TAS were below the critical level in Dhading and Mahottari districts, the current CFA prevalence revealed high whereas in Bara and Lalitpur districts, it is still below the critical level, i.e., < 2% (Fig. 2).

## Discussion

WHO launched the global program to eliminate lymphatic filariasis in 2000 with the elimination target globally by 2020 and later on it was extended up to 2030 [16]. Preventive chemotherapy using a mass annual single dose of DEC and albendazole is the main transmission control strategy [21] in preventing new infections thereby achieving elimination. All three filarial parasites viz., *W. bancrofti, B. malayi,* and *B. timori* with periodic and sub-periodic physiological races are known to respond well to the drugs used in the program. About 1380 million people were at risk of infection in 72 countries which were earlier known to be endemic [22]. Ten countries were classified as non-endemic with no evidence of indigenous transmission. Fifty-eight countries have successfully interrupted the transmission and are under post-MDA surveillance [22]. When the program was launched, guidelines for program planning and implementation [23] and monitoring and evaluation, particularly on the decision-making were available. However, tools and protocols for monitoring and evaluation [24] were not operationally feasible with highly conservative levels of the threshold. Hence based on inputs from the researchers, the program was scaled up and revised protocol [25–31], in 2011 Transmission Assessment Survey (TAS) was developed and recommended for monitoring and evaluation of the LF elimination program [32]. The immunochromatographic test-based tools have been recommended for assessment and verification of the absence of new infection during the post-MDA period for detecting CFA.

The Nepal government had started the MDA program with DEC and albendazole in presently studied Terai districts (Bara and Mahottari), and hilly districts (Dhading) in 2007 while in Lalitpur district in 2010 [20]. Nepal government has completed pre-TAS, TAS I, and TAS II in 2013, 2014, and 2017–2018, respectively, in the sentinel sites of those districts. These sentinel sites are considered hotspot regions. But there are certain demerits of the Transmission Assessment Survey (TAS), which is carried out in a school children-based model instead of a community-based one. In the majority of the districts, the MDA program was stopped based on antigenemia prevalence < 2% (critical level) which may not always reflect the accurate antigenemia prevalence at the community level [33]. Our study indicated a high prevalence of CFA in one of each hotspot area of Mahottari district (Terai region) and Dhading district (hilly region) of Central Nepal. High CFA prevalence in these districts was found well correlated with the high baseline prevalence as well as less number of MDA rounds covered. Endemic areas with high baseline infection levels will require more sustained MDA interventions [34]. The required number of MDA rounds depends on the baseline prevalence of the infection [35].

In the Bara district of the Terai region, 11 rounds of MDA were extended due to the higher antigenemia prevalence in both TAS I and TAS II compared to the baseline prevalence. Sustained extensive MDA round was found well correlated with the reduced CFA prevalence despite the less IQR and median treatment coverage; whereas, in the Mahottari district of the same region and Dhading district of the hilly region, CFA prevalence in TAS I showed drastically reduced compared to the baseline prevalence. Hence MDA was stopped after six rounds of MDA in 2014. TAS II was assessed in 2017 which also showed CFA prevalence below the critical value and MDA intervention was not extended. High baseline prevalence, demerits of the TAS assessment, and reduced MDA rounds in Mahottari and Dhading districts enhanced the spread of the residual LF infection in the community which is the indicative of high prevalence of CFA in the present study.

In the Lalitpur district of the hilly region, eight rounds of MDA continued till 2017 due to the high IQR. There was a large variation in treatment coverage among all eight rounds of MDA intervention. Out of six rounds of MDA intervention, only two rounds of intervention met the WHO guideline of 65% treatment coverage. Despite the reduced antigenemia prevalence in all Transmission assessments compared to the baseline prevalence, the extension of MDA rounds up to eight impacts reducing the current CFA prevalence. Only one positive case of CFA was observed which was also not the local origin but imported from another endemic Banke district of the Terai region.

Despite the proven effectiveness of the MDA drugs, [36] we were not speechless by the persistent increase in CFA prevalence in hotspots districts. Based on WHO guidelines, a minimum of five rounds of MDA may be sufficient to interrupt the transmission of LF [37–39]. However, our studies showed that in areas with high baseline antigenemia prevalence, five to six rounds of MDA intervention [40] are not sufficient to interrupt the transmission.

The success of the MDA program relies on sufficient treatment coverage and optimal drug uptake [41, 42]. The minimum effective treatment coverage of the total population was estimated to be 65% [43]. Although five rounds of MDA have been recommended for the minimum target population, it depends on the baseline prevalence of infection, initial intensity of transmission, the efficacy of drugs, and other factors [44–49].

CFA prevalence above the 1% level in 2–8 years old children is indicative of the existence of foci of persistent infection and transmission of LF in the community [50]. These areas require focus during post-MDA surveillance to detect the signals for a possible resurgence of infection.

## Conclusions

The cluster of antigen-positive cases at the community level could lead to the risk of a resurgence of new infections. Analysis of LF infection indices in children born after the MDA program is crucial to assess the LF infection resurgence in infection persistence hotspots. Antigenemia survey among the children who have never participated in MDA intervention in hotspots of four endemic districts of Central Nepal revealed infection resurgence in two districts. Infection resurgence in these districts was found well correlated with the high baseline prevalence and low MDA rounds. High treatment coverage and assessment of antigenemia below critical value in sentinel sites are not only sufficient to decide whether to interrupt or eliminate LF infection at the community level. Re-assessment of the infection and extensive rounds of focal MDA interventions are required to stop the resurgence of the new infection to achieve the elimination goal.

### Supplementary Information

**Additional file 1: Figure S1.** A positive test read at 10 min.

## Data Availability

All relevant data supporting the conclusions of this article are included in the article.

## Abbreviations

LF: Lymphatic filariasis
MDA: Mass drug administration
CFA: Circulating filarial antigen
DHS: Department of health services
MF: Microfilaria
Ag: Antigen
ICT: Immunochromatographic test
TAS: Transmission assessment survey
WHO: World Health Organization
GPELF: Global Program to Eliminate Lymphatic Filariasis
