# Supplementary figures and images for: Assessment of antigenemia among children in four hotspots of filarial endemic districts of Nepal during post-MDA surveillance
Source: Trop Med Health. 2023 Aug 24;51:47. doi: 10.1186/s41182-023-00538-4 (PMC10464004; doi:10.1186/s41182-023-00538-4)

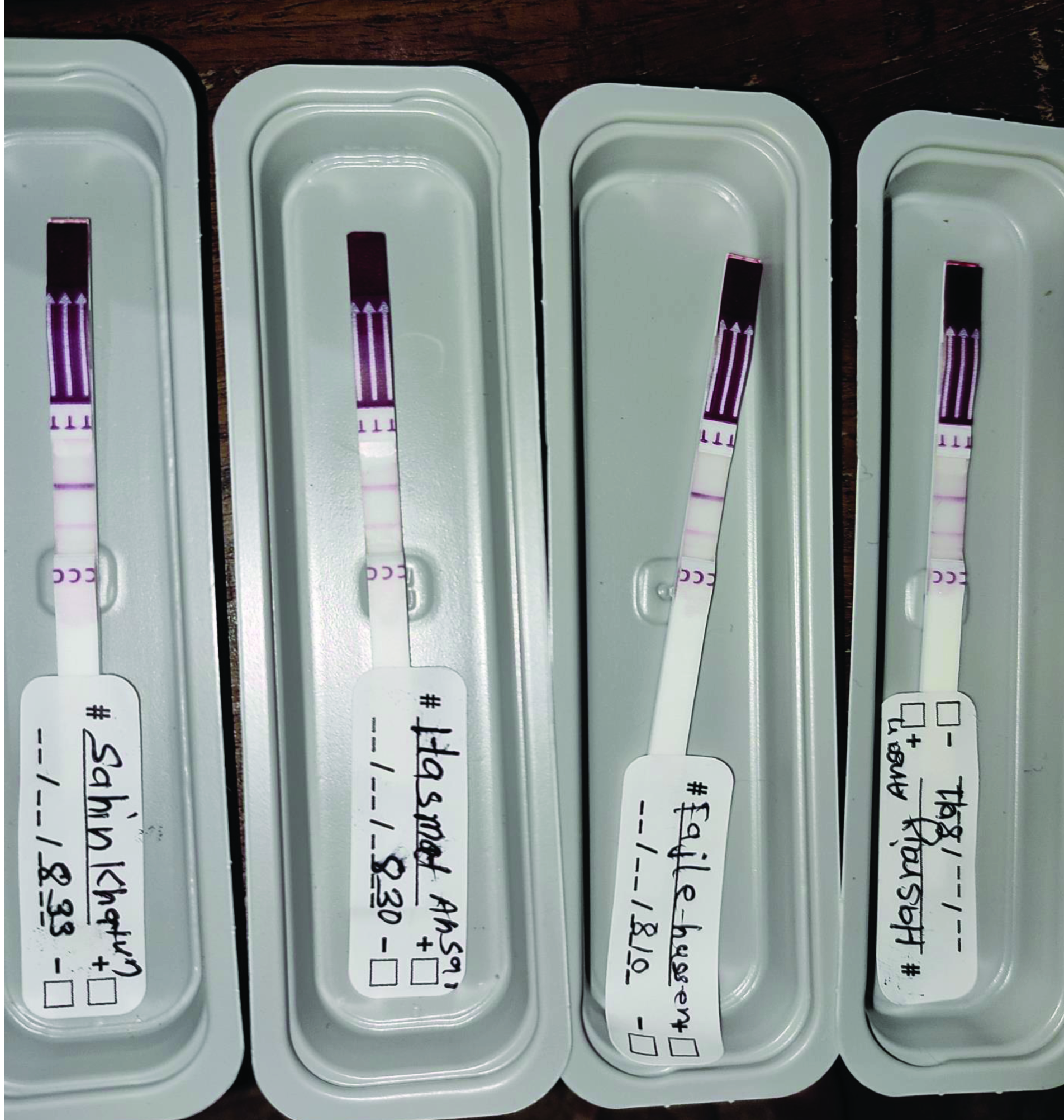

Supplement: Supplementary file 1 — Additional file 1: Figure S1. A positive test read at 10 min. [file 41182_2023_538_MOESM1_ESM.tif]
